# Editorial: COVID-19 and Venous Thromboembolism

**DOI:** 10.3389/fcvm.2021.818231

**Published:** 2021-12-20

**Authors:** Yugo Yamashita

**Affiliations:** Department of Cardiovascular Medicine, Graduate School of Medicine, Kyoto University, Kyoto, Japan

**Keywords:** COVID-19, venous thromboembolism (VTE), pulmonary embolism, deep vein thrombosis (DVT), anticoagulant

In the current special issue of Frontiers in Cardiovascular Medicine, the latest original data of venous thromboembolism (VTE) related to coronavirus disease 2019 (COVID-19) from all over the world have been reported as well as some domestic issues in each country. The COVID-19 has become a huge threat all over the world as a pandemic, which also could cause VTE, including pulmonary embolism (PE). However, there is still limited data on this important topic, and the optimal strategies for the prevention of VTE in COVID-19 still remain unknown. Further basic and clinical studies would be important to enhance the knowledge of COVID-19 and VTE.

COVID-19 is a viral illness caused by severe acute respiratory syndrome coronavirus 2 (SARS-CoV-2), the main pathophysiology is a respiratory infectious disease. Coagulopathy in patients with COVID-19 has been reported, which could lead to thromboembolic complications (1). Although detailed pathophysiology of coagulopathy in patients with COVID-19 is still unknown, hemostatic assays could be clinically important for understanding of development of VTE. The usefulness of viscoelastic testing with rotational thromboelastometry has been reported by Hulshof et al.. They found interesting results revealing that rotational thromboelastometry tests in patients in intensive care unit with COVID-19 showed hypercoagulability and severe hypofibrinolysis persisting over at least 6 weeks.

Considering the high risk of VTE in COVID-19 patients, it could be important for clinicians to conduct an appropriate imaging examination of these patients when they are suspected of VTE during the course of COVID-19 treatment to avoid under-diagnosis of VTE. However, a previous study reported that only a small number of patients were evaluated with contrast-enhanced computed tomography (CT) examination in real-world clinical practice, which suggests some reluctance to conduct imaging examinations due to the risk of infection to healthcare providers (2). Thus, practical diagnostic strategies for VTE could be clinically relevant. However, there is a lack of explicit indications regarding the best algorithm for diagnosing VTE, and it is not clear how to identify subjects who should undergo contrast-enhanced CT examinations. To assess the indication of contrast-enhanced CT examinations according to guideline-recommended algorithms for diagnosing PE, a retrospective analysis of a cohort of patients hospitalized for COVID-19 and acute respiratory failure has been reported by Porfidia et al.. They interestingly showed that COVID-19 patients with acute respiratory failure underwent imaging examinations without an established criterion although the diagnosis of PE was not rare with contrast-enhanced CT examinations, suggesting the need for validated tools for identifying COVID-19 patients who require contrast-enhanced CT examinations for suspected PE.

Although VTE related to COVID-19 has been clinically relevant, a constant increase of PE hospitalizations not related to COVID-19 was reported (3). To investigate the potential contributors to the observation, valuable consecutive cases with a hospital admission using claims data in Germany have been reported by Husser et al.. They showed that there was an increase in PE cases since early May 2020 compared to corresponding periods in 2016–2019, and this surplus was significant even when controlling for changes in potential modulators such as demographics, volume depletion, non-COVID-19 pneumonia, contrast-enhanced CT use, and preceding COVID-19 infections.

The prevalence of VTE varied widely among these reports, which might be due to the different study populations, different diagnostic strategies, and different thromboprophylaxis management (4). Furthermore, ethnic differences and distinct resource availability may have notable implications in the presentation and diagnosis of VTE. Epidemiological data of COVID-19-associated thrombosis in the Japanese population and variant strains of SARS-CoV-2 have been reported by Oba et al.. The patients with COVID-19 admitted to a university hospital in Japan were retrospectively analyzed. They revealed relatively lower incidences of VTE and higher incidences of arterial thrombosis which might be a unique feature in the Asian population. Further large-scale studies would be required to establish appropriate management strategies in each region and ethnicity.

Overall, the current various original articles give a valuable insight for better understanding and future perspective of COVID-19 and VTE. The topic editors strongly wish that the current special issue of the Research Topic: *COVID-19 and Venous Thromboembolism* could be helpful for healthcare providers as well as patients with COVID-19 all over the world.

## Author Contributions

YY fulfills the standard requirements for authorship and drafted the manuscript.

## Conflict of Interest

The author declares that the research was conducted in the absence of any commercial or financial relationships that could be construed as a potential conflict of interest.

## Publisher's Note

All claims expressed in this article are solely those of the authors and do not necessarily represent those of their affiliated organizations, or those of the publisher, the editors and the reviewers. Any product that may be evaluated in this article, or claim that may be made by its manufacturer, is not guaranteed or endorsed by the publisher.
